# Immunotherapy in Non-Small-Cell Lung Cancer: A Modified Delphi Survey Consensus on First Line Treatment, Special Populations and Rechallenge

**DOI:** 10.3390/biomedicines12122742

**Published:** 2024-11-29

**Authors:** Francesca Colonese, Alessandra Bulotta, Carlo Genova, Diego Signorelli, Laura Bonanno, Claudia Proto, Immunotherapy in NSCLC Delphi Panel, Diego Luigi Cortinovis

**Affiliations:** 1 Medical Oncology Unit, Fondazione IRCCS San Gerardo dei Tintori di Monza, 20900 Monza, Italy; diegoluigi.cortinovis@irccs-sangerardo.it; 2 Department of Oncology, IRCCS Ospedale San Raffaele, Via Olgettina 60, 20132 Milan, Italy; bulotta.alessandra@hsr.it; 3 Department of Internal Medicine and Medical Specialties, University of Genoa, 16132 Genoa, Italy; carlo.genova@hsanmartino.it; 4 Academic Medical Oncology Unit, IRCCS Ospedale Policlinico San Martino, 16132 Genoa, Italy; 5 Niguarda Cancer Center, Grande Ospedale Metropolitano Niguarda, 20162 Milan, Italy; diego.signorelli@ospedaleniguarda.it; 6 Department of Surgery, Oncology and Gastroenterology, University of Padova, 35131 Padova, Italy; laura.bonanno@iov.veneto.it; 7 Medical Oncology 2, Istituto Oncologico Veneto IOV IRCCS, 35128 Padova, Italy; 8 Medical Oncology Department, Fondazione IRCCS Istituto Nazionale Tumori, 20133 Milan, Italy; claudia.proto@istitutotumori.mi.it; 9 Department of Medicine, University of Milano-Bicocca, 20126 Monza, Italy

**Keywords:** non-small-cell lung cancer, immunotherapy, Delphi survey, immune checkpoint inhibitors

## Abstract

**Background**: The treatment landscape for non-small cell lung cancer (NSCLC) has evolved significantly with the advent of immunotherapy. Nonetheless, uncertainty regarding optimal first-line treatments, special populations, and the feasibility of rechallenge remains. This study aims to investigate Italian oncologists’ opinions on these aspects through a Delphi Survey. **Methods**: A steering committee (SC) of six oncologists identified three topics of interest, namely NSCLC (first line) therapeutic choice, NSCLC special populations, and NSCLC immunotherapy rechallenge), and drafted several topic-related statements to be voted in the Delphi Survey by the 61 oncologists forming the Delphi Panel. The survey included two rounds, wherein the experts rated their agreement/disagreement with the statements on a 5-point Likert scale. Consensus was defined as agreement/disagreement by at least 75% of the panel. **Results**: The SC drafted 69 statements for the first round, of which 16 (23.2%) met the agreement threshold, 5 (7.2%) met the disagreement threshold, and 48 (69.6%) did not reach consensus. The SC revised the latter statements and drafted 37 for the second round. Overall, 5 (13.5%) statements met the agreement threshold, 1 (2.7%) met the disagreement threshold, and 31 (83.8%) did not reach consensus in the second round. **Conclusions**: The survey showed agreement on the necessity of molecular characterization, mutations, smoke, the role of steroid therapy, and immunotherapy rechallenge, and revealed several areas of uncertainty among Italian oncologists on the use of immunotherapy in NSCLC. Statements—where consensus was not met—can be used to guide future clinical research in resolving the issues.

## 1. Introduction

Approximately 85% of lung cancer diagnoses belong to the non-small-cell lung cancer (NSCLC) subtype, and over half of newly diagnosed NSCLC patients present with stage IV disease [1,2,3].

Immunotherapy strategies based on programmed cell death protein 1 (PD-1), PD-1 ligand (PD-L1), and cytotoxic T-lymphocyte antigen 4 (CTLA-4) immune checkpoint inhibitors (ICIs) have been widely investigated in the last decade, representing substantial progress in the treatment of advanced NSCLC with respect to the traditional platinum-based chemotherapy (CT) regimens [4,5]. In particular, single-agent pembrolizumab (anti-PD-1) was the first to significantly prolong the progression-free survival (PFS) and overall survival (OS) compared with platinum-based CT in patients with advanced NSCLC and PD-L1 expression ≥50% in the KEYNOTE-024 and KEYNOTE-042 trials, becoming a standard of care in the first-line setting [6,7,8]. More recently, other ICI monotherapies have been added to the available first-line treatments in Europe, atezolizumab (anti-PD-L1) in patients with PD-L1 expression ≥50% on tumor cells (Impower110 trial) [9,10], and cemiplimab (anti-PD-1) in those with PD-L1 expression ≤50% (Empower-lung 1 trial) [11] The improved efficacy in terms of OS of immunotherapy plus doublet CT over the single ICI treatment was reported in both squamous (KEYNOTE-407 and IMpower131) and non-squamous (Keynote-189, IMpower 150, IMpower 130, and IMpower132) NSCLC [12,13,14,15,16,17].

The combination of distinct ICIs with complementary mechanisms of action was also evaluated. The phase III CheckMate 227 study showed that nivolumab plus ipilimumab (anti-CTLA-4) treatment was superior to CT alone in chemo-naïve NSCLC patients [18]. The phase III study CheckMate 9LA investigated the effect of the addition of two CT cycles to nivolumab plus ipilimumab, reporting the survival benefit of this therapeutic regimen compared with CT alone, regardless of the PD-L1 expression [19]. This evidence suggests the improved efficacy of ICI combination in NSCLC.

Despite these advancements in NSCLC treatment, many “gray areas” regarding the best therapeutic approach according to various clinical situations remain and are debated among oncologists. The response to immunotherapy is often not maintained over time, and around 20% of patients do not respond to ICIs; to date, there are no markers—apart from tissue PD-L1 expression—that can guide patient selection [20,21,22]. However, there are a series of clinical factors stemming from the analysis of studies, subgroups, inclusion/exclusion criteria, meta-analysis, etc., that are routinely used in clinical practice to best choose which treatment to provide to the patient [23,24]. In the absence of exhaustive data, the identification and discussion of such gray areas are of utmost importance in resolving them through future investigations and in advising clinical practice [25,26].

A consensus exercise through the use of the Delphi Survey methodology [27] among Italian oncologists was thus carried out, with the following aims: (a) highlight such major areas of uncertainty in the field of NSCLC immunotherapy treatment; (b) identify clinical, biomolecular and response parameters actually taken into account by clinicians in daily practice; and (c) describe and confront the level of agreement or disagreement among Italian, NSCLC expert clinicians, and summarize a consensus where possible. In Italy and all of Europe, at the time of this study, the ICIs pembrolizumab and atezolizumab, or chemotherapy alone, based on histology, were approved for patients with advanced NSCLC and PDL1 > 50%. Possible combinations of chemotherapy + immunotherapy included pembrolizumab + double platinum-based chemotherapy (depending on histology), nivolumab + ipilimumab + double platinum-based chemotherapy (depending on histology), or chemotherapy alone. All approved treatments were reimbursed in public health institutes in Italy. All available diagnostic panels were reimbursed for metastatic NSCLC.

Here, we report our findings in accordance with the ACCORD guidelines on consensus studies [28].

## 2. Methods

### 2.1. Steering Committee and Delphi Panel

A steering committee (SC) was formed in December 2022. It included six nationally recognized medical oncologists with deep expertise in clinical management and research of NSCLC—namely AB, CG, DS, LB, CP, and FC. FC served as chairwoman and project coordinator. All SC members work in relevant Italian oncological hub centers for NSCLC. One additional member of the SC (FP) served as a methodologist and facilitator to support the other SC members through meeting facilitation, material preparation, and survey management. Through a non-systematic review of the scientific literature on NSCLC and immunotherapy and internal discussions, the SC identified three topics of interest: (1) NSCLC (first-line) therapeutic choice, (2) NSCLC special populations, and (3) NSCLC immunotherapy rechallenge. The SC then generated some topic-related statements to be used in the first round of voting of the Delphi Survey by the Delphi Panel.

The Delphi Panel (i.e., those responding to the Delphi Survey) was formed by 61 oncologists working in Italy with proven expertise in NSCLC immunotherapy management. The SC recruited the panelists through personal invitations by email, telephone, or other means. The panelists were allowed to suggest other participants to the SC but not to invite them directly. The recruiting process was overseen by the methodologists and the SC chairwoman to keep track of all participants and avoid double invitations.

### 2.2. Modified Delphi Survey

A Delphi survey is a structured and common method used to gather and consolidate expert opinions on particular topics or issues where empirical evidence is scarce or cannot be collected. The process involves multiple rounds of surveys or questionnaires answered by a panel of experts called the “Delphi Panel”. The iterative nature of the process allows for the refinement of opinions over multiple rounds, and it helps to identify areas of consensus and disagreement among the experts. In particular, the Delphi Survey is used to quantify consensus through the evaluation of the level of agreement and to develop consensus through the resolution of differences of opinion [27].

Instead of implementing several survey rounds until a final consensus was reached, as contemplated by the original methodology, the process was adapted by carrying out only two rounds of voting. This was conducted because consensus was often considered impossible to reach and to avoid the project being dragged for too long, thus resulting in participants losing interest and dropping out. After the first voting round, three meetings between the SC and some Delphi panelists (around 20 for each meeting) were used to discuss the results, especially those statements that did not reach the consensus threshold; the panelists were different at every meeting such that after the three meetings all panelists had participated to the discussion and provided their feedback. These inputs were used by the SC to reformulate the statements that did not reach consensus. The revised statements were then implemented in the second round of voting. Eventually, the SC discussed the full results from the Delphi Survey and used them to draft the present paper.

The Delphi Panel voted on the statements using a 1–5 Likert scale (1 = total disagreement; 2 = partial disagreement; 3 = doubtful; 4 = partial agreement; 5 = total agreement). Before voting, panelists were presented with the project and its objectives, but no additional scientific materials or information was provided, as one of the aims of the survey was to investigate the clinical practice normally carried out by the clinicians. The SC did not vote for the Delphi Survey, and Delphi panelists—apart from being granted the “group authorship” of this paper—received no further incentives for participating. No open answers were allowed, and panelists were not asked to explain their responses. The statements reached a consensus on agreement when ≥75% (i.e., three-quarters) of the Delphi Panel voted either partial agreement or total agreement. The statements reached consensus on disagreement when ≥75% (i.e., three-quarters) of the Delphi Panel voted either partial disagreement or total disagreement. These percentages are in accordance with the indication of the Italian Ministry of Health for consensus methodologies [29]. The voting was carried out online through the use of the online survey platform SurveyMonkey. The results were collected by the methodologist, who merged and anonymized the data before sharing it with the other SC members.

All meetings, discussions, surveys, etc., were carried out in Italian. The study was not prospectively registered.

### 2.3. Definitions

High disease burden was defined by the sum of the longest diameter of target lesions ≥10 cm or a primary tumor with a diameter of ≥10 cm, an increase in LDH of ≥2 ULN, presence of hepatic involvement, lymphangitis, pericardial effusion, brain involvement that cannot be addressed with local techniques, or an oncological emergency [30]. Patients with PS ECOG 0-1 without significant comorbidities were considered “fit”.

## 3. Results

### 3.1. First Round

The SC drafted 69 statements for the first round, of which 32 (46.4%) were on therapeutic choice, 15 (21.7%) were on special populations, and 22 (31.9%) were on immunotherapy rechallenge. In the first round, 60 out of 61 (98.4%) panelists voted. Of the 32 statements on therapeutic choice, 7 (21.9%) reached consensus on agreement, 3 (9.4%) reached consensus on disagreement, and 22 (68.8%) did not meet the consensus threshold (Table 1). Of the 15 statements on special populations, 2 (13.3%) reached consensus on agreement, and 13 (86.7%) did not meet consensus; no statement reached consensus on disagreement (Table 2). Of the 22 statements on immunotherapy rechallenge, 7 (31.8%) reached consensus on agreement, 2 (9.1%) reached consensus on disagreement, and 13 (59.1%) did not reach consensus. Overall, 16 (23.2%) statements met the agreement threshold, 5 (7.2%) met the disagreement threshold, and 48 (69.6%) did not reach consensus (Table 3).

### 3.2. Second Round

The SC revised and unified the 48 statements which did not reach consensus and eventually drafted 37 statements to be voted on in the second round, namely 22 (59.5%) on therapeutic choice, 5 (13.5%) on special populations, and 10 (27.0%) on immunotherapy rechallenge. In the second round, 58 out of 61 (95.1%) panelists voted. Of the 22 statements on therapeutic choice, 2 (9.1%) reached consensus on agreement, 1 (4.5%) reached consensus on disagreement, and 19 (86.4%) did not meet the consensus threshold (Table 1). Of the five statements on special populations, 1 (20.0%) reached consensus on agreement, and 4 (80.0%) did not meet consensus; no statement reached consensus on disagreement (Table 2). Of the 10 statements on immunotherapy rechallenge, 2 (27.0%) reached consensus on agreement, and 8 (80.0%) did not reach consensus; no statement reached consensus on disagreement (Table 3). Overall, 5 (13.5%) statements met the agreement threshold, 1 (2.7%) met the disagreement threshold, and 31 (83.8%) did not reach consensus.

## 4. Discussion

This consensus study addressed the recent and fast-evolving setting of immunotherapy in NSCLC, highlighting the presence of several areas of uncertainty regarding the best clinical practice to carry out according to various factors.

### 4.1. Therapeutic Choice and First-Line Treatment

The panelists unanimously agreed on the necessity of molecular characterization of the tumor, possibly with NGS, in order to maximize the therapeutical choice by (1) excluding some of the main oncogene-addicted tumors from ICI employment; (2) guiding the choice of non-targeted systemic treatment in the first line; and (3) guiding the type of monitoring to be performed during the first-line treatment. The introduction of such molecular diagnostic examinations will also allow an increase in the number of genes and associated alterations explored in clinical practice, resulting in a deeper understanding of molecular features, which will, in turn, be useful in predicting response to treatment, the occurrence of primary and secondary resistance, and possibly the development of new targeted drugs [23,31].

### 4.2. Immunotherapy and Chemoimmunotherapy

Our survey investigated clinicians’ preferences toward the use of (chemo)immunotherapy therapies. Available chemoimmunotherapy treatments comprise triplet and quadruplet regimens. Both include the use of two chemotherapeutics, namely a platinum-based one and an antifolate antineoplastic agent, usually pemetrexed, in case of adenocarcinoma histology, or a taxane in case of squamous histology. CT is combined with pembrolizumab or ipilimumab and nivolumab for triplet and quadruplet regimens, respectively.

The Panel agrees that, in the case of patients with PD-L1 ≥ 50% and high disease burden, chemoimmunotherapy rather than just immunotherapy should be considered as the first treatment choice, despite this not being possible in Italy to date due to reimbursement limitations. The reason behind this preference might lay in the fact that, although chemoimmunotherapy is not associated with an overall benefit over immunotherapy alone, it seems associated with an early survival advantage, even in patients with PD-L1 expression ≥ 90% [32], and seems more effective against brain metastases, although the quality of evidence on brain metastasis is still low [33].

On the other hand, in case of patients with PD-L1 < 50% already candidates for chemoimmunotherapy, harbored mutations, disease burden, and the possibility of a platinum-based second-line treatment or immunotherapy rechallenge do not single-handedly direct towards the use of triplet or quadruplet regimens, according to the experts. Of note, consensus was almost reached on the topic of using triplet regimens (CT + pembrolizumab) in those with PD-L1 < 50% and *KRAS* (G12C), *MET*, *RET*, *HER2*, or *NTRK* mutations, possibly to be able to carry out more CT cycles if needed. Despite no clear consensus—consistent with the absence of clear, supportive data—in the presence of a molecular driver, the Panel tendency is to complete the four CT cycles, probably due to the belief that CT contribution is particularly relevant in these cases.

### 4.3. The Role of Mutations

Our survey addressed several mutations and their role in contributing to the first-line treatment choice. In particular, we focused on *MET*, *BRAF* (V600), *RET*, *HER2*, *KRAS,* and *NTRK*, which are included in the main common NGS panel employed routinely in clinical practice; *EGFR*, *ALK,* and *ROS* were not included in the survey as their presence excludes the use of first-line immunotherapy-based combinations and guides treatment towards targeted therapies [34].

*KRAS* mutations are among the most commonly found in NSCLC (around 30% of all cases) [31], with the G12C alteration being the most frequent (40% of all *KRAS* mutations); they are often associated with high PD-L1 expression and higher tumor mutational burden and are mostly considered a poor prognostic biomarker [35,36]. A few randomized clinical trials (RCTs) and observational studies have shown immunotherapy to be valid in this setting. First-line pembrolizumab, whether alone or associated with CT, seems to be superior to CT alone regardless of *KRAS* status, as suggested by post-hoc analyses of the KEYNOTE-042 and -189 studies [37]. According to the Panel, when a *KRAS* mutation is detected, the first line treatment choice should be immunotherapy in patients with PD-L1 ≥ 50% and chemoimmunotherapy in those with PD-L1 < 50%. This is based on the fact that at least three meta-analyses have reported higher survival benefits with immunotherapy and chemoimmunotherapy over CT alone [38,39,40], and a large retrospective study in more than 1100 patients with PD-L1 > 50% and *KRAS* mutations reports longer OS compared to wild-type patients when ICI monotherapy rather than ICI-CT combination is used [41], a finding supported by others [42]. However, *STK11* ± *KEAP1* co-mutations with *KRAS* are related to immunotherapy resistance and a lower proportion of PD-L1 ≥ 50% expression [43,44]. Studies on therapeutic choices in the case of *KRAS* co-mutations STK11 ± KEAP1 are still limited, and the Panel could not reach a consensus. Preliminary data published after the completion of our survey suggest that patients with *KRAS* mutated and PD-L1-negative NSCLC treated with (chemo)immunotherapy experience worse OS, with this association being largely driven by STK11 and KEAP1 co-mutation, which are enriched in PD-L1 negative NSCLC; indeed, patients with triple *KRAS*, *KEAP*, and *STK11* mutations and no PD-L1 expression experienced the worst outcomes [45]. On the other hand, *KRAS* and *TP53* co-mutation increase response rate and survival to first-line pembrolizumab in NSCLC with PD-L1 ≥ 50% compared to wild-type *KRAS* patients [46].

Data on several other mutated NSCLC groups are still mostly inconclusive; some findings seem to suggest some benefits with ICIs in the case of pre-treated patients with *HER2*, *BRAF*, *RET*, and *MET* mutations, similar to those observed in unselected patients [47,48], while other studies suggest a poor benefit from ICIs in both pre-treated [49] and treatment-naïve patients [50]. According to the Panel, immunotherapy must not be considered as a first option when treating *RET*- (and *NTRK*-) mutated NSCLC, even when PD-L1 ≥ 50%, as targeted therapies result in better outcomes [50,51]; on the other hand, *MET*, *BRAF* (V600), and *HER2* mutations do not have a role in directing first-line treatments towards ICI monotherapy or chemoimmunotherapy, regardless of PD-L1 expression.

### 4.4. The Role of Smoking

Regarding smoking, the Panel agrees that: (1) in case of never smokers with non-oncogene-addicted NSCLC and PD-L1 ≥ 50%, first-line treatments should rely on chemoimmunotherapy, although this not being pursuable in Italy to date due to regulatory reasons; (2) in case of never smokers with non-oncogene-addicted NSCLC and PD-L1 ≥ 90%, CT should never be used alone, and that first-line treatment should be immunotherapy; however, it would consider first-line chemoimmunotherapy if this was possible regulatory-wise. Reasons for this are related to the fact that, although ICIs are effective in case of high PD-L1 expression, they are less effective in never smokers, where CT seems to exert more benefits, as shown in several studies. In a case-control matched analysis by Cortellini et al., current/former smokers with metastatic NSCLC patients and PD-L1 expression ≥ 50% receiving first-line pembrolizumab experienced improved PFS and OS, whilst the opposite trend was found within NSCLC patients treated with first-line platinum-based CT [52]. In the KEYNOTE-189 trial, the addition of immunotherapy benefited smokers more than never-smokers [12,53]. Prolonged survival in smokers with NSCLC undergoing immunotherapy or chemoimmunotherapy was confirmed by two meta-analyses involving >10,000 [54] and >14,000 [55] patients. Indeed, smoking seems associated with changes in the tumor microenvironment favoring response to ICIs [56,57]. In particular, it could cause mutations in *POLD1*, *POLE*, and *MSH1* genes, resulting in a high neoantigen burden [58] and supporting the activation and recruitment of macrophage M1 and inflammatory T cells [56].

### 4.5. The Role of Concurrent Steroid Therapy

Among the several concurrent treatments that can affect response to ICIs [59,60], our survey focused on steroid therapy. The Panel agrees that in patients with PD-L1 ≥ 50% under steroid treatment (prednisone 10 mg/day or equivalent regimes). However, immunotherapy is the recognized first-line treatment; the panel would consider combining ICI with CT if it was possible regulatory-wise. Ricciuti et al. demonstrated that patients receiving ≥10 mg/day of prednisone when starting ICI treatment had shorter survival outcomes than those receiving 0 to <10 mg/day. However, subsequent analysis by reason for corticosteroid administration showed significantly worse survival outcomes only among patients who received ≥10 mg/day prednisone for cancer-related indications compared with those receiving prednisone (even ≥10 mg/day) for cancer-unrelated reasons [61]. The association between baseline steroids administered for cancer-related indications and worse clinical outcomes with PD-1/PD-L1 checkpoint inhibitors is supported by other studies showing a significantly higher risk of disease progression and death in patients receiving steroids due to palliative reasons [59,62]. Thus, this difference seems to be driven by a poor-prognosis subgroup of patients who receive corticosteroids for palliative indications rather than corticosteroids affecting ICI response [59,61]. Clinicians should be aware of these considerations and continue steroid therapy during ICI treatment if deemed necessary [63].

### 4.6. Special Populations

Our survey addressed the topic of the best immunotherapy regimen in many special populations, such as patients suffering from autoimmune disease, hepatic and brain metastasis, those of advanced age, with poor performance status, high-disease burden, etc.

Regarding active autoimmune diseases, there is no absolute contraindication to the use of CT-ICI provided that high doses of steroids are not used (see Section 4.5); in this perspective, selective immunosuppressants instead of steroids should be considered [63]. According to the Panel, in those patients with PD-L1 < 50% and suffering from comorbidities that can affect the use of multiple CT cycles, the first-line treatment should combine ipilimumab with nivolumab and two CT cycles. In case of autoimmune conditions, whenever feasible, combining CT with only one ICI is considered preferable to the combination of short-course CT with two ICIs, possibly because of immunotherapy-related adverse events (irAEs) and CT immunosuppressive properties [64,65].

Several factors can be related to worse outcomes from ICIs. These include the Lung Immune Prognostic Index (LIPI) score, combining derived neutrophils/(leukocytes minus neutrophils) ratio (dNLR) >3 and lactate dehydrogenase (LDH) level greater than upper limit of normal [66], the presence of liver or brain metastases [19,67], disease burden-induced ECOG performance status 2 [68,69] and advanced age (>75 years old) [19]. Patients with untreated brain metastases have been generally excluded from RCTs. A pooled analysis of CT plus pembrolizumab combination trials in the first line, enrolling both patients with previously treated brain metastases and patients with untreated asymptomatic brain metastases with diameter ≤ 1.5 cm and off steroids, showed a survival benefit for the combination in comparison to CT alone. However, no intracranial efficacy data have been collected [70]. In the CheckMate 9-LA study, brain metastases were not considered an exclusion criterion if adequately treated, asymptomatic for 2 weeks or more, and with corticosteroids permitted if the dose (10 mg daily prednisone or equivalent) was stable or decreasing for at least 2 weeks before starting the study treatment. An exploratory analysis of intracranial efficacy showed a benefit for CT plus ICIs combination in terms of OS, intracranial PFS, response rate, and time to development of new brain lesions [71]. Despite these data, the experts could not meet consensus on the use of triplet or quadruplet regimens according to the presence of hepatic/brain metastasis, LIPI and LDH score, advanced age, performance status = 2, non-adenocarcinoma histology, negative PD-L1, and high disease burden, suggesting that subgroup analyses and these features are not independent factors guiding treatment choice. However, according to the panel, the co-presence of negative PD-L1 and non-adenocarcinoma histology favors the use of the quadruplet regimen compared to the triplet regimen, while other oncologists support the use of CT plus double ICI based on negative PD-L1 expression only, regardless of histology [72]. Reasons for this might be due the fact that subgroup analyses of the 5-year follow-up of the KEYNOTE-407 did not detect a significant survival benefit from CT plus pembrolizumab in patients with advanced squamous NSCLC and PD-L1 expression < 1% [73]; on the contrary, these patients seem to benefit the most from the combination of CT plus nivolumab and ipilimumab [19,74]. In the CheckMate 9LA, nivolumab and ipilimumab combined with CT showed an improvement in median OS versus CT in the subgroups of patients with PD-L1-negative (17.7 vs. 9.8 months), non-squamous (17.8 vs. 12 months) and squamous histology (14.5 vs. 9.1 months), and 12-month OS rates were respectively 63% and 47% in the PD-L1 negative subgroup [19]. After 2 years of follow-up, the OS benefit of the quadruplet regimen remained significant compared to CT alone, even for patients with PD-L1 expression < 1% [74].

### 4.7. Immunotherapy Rechallenge

In the first studies on ICI treatment, immunotherapy used to be carried on until unacceptable toxicity or disease progression but has now been limited to a 2-year period as available data suggest that treatments of fixed duration of at least 12 months provide durable benefits [21,75,76]. This is possibly due to the fact that immunotherapy induces polyclonal and memory-adaptive antitumor immunity to control the clonal heterogeneity of the disease and reset the tumor–host immune interaction [77]. Case reports and observational studies, despite not providing a high level of certainty, seem to show benefits in terms of PFS in patients with partial response (PR), complete response (CR), or treatment-related adverse events at the time of ICI discontinuation [21,75,78,79,80,81]. When asked when to discontinue ICIs, the Panel agreed to discontinue in case of CR but could not meet a consensus in case of PR or stable disease (SD). Lifelong ICI treatment, on top of being economically unsustainable, seems to be unnecessary and sometimes harmful; conversely, ICI discontinuation in the case of PR/CR after 2 years is not globally accepted yet [21], as also shown by our results.

Despite the ICI response being durable following discontinuation, disease progression can also occur (possibly due to the loss of PD-1/PD-L1 inhibition caused by a rapid antibody clearance or resistance due to removal of the PD-1/PD-L1 blockade) [82], highlighting the need for eventually resuming immunotherapy. ICI rechallenge following progressive disease seems to result in clinical benefits in up to 70% of cases, especially if patients carried out immunotherapy for at least 1 year and experienced a response to the initial treatment [83,84]. Regarding immunotherapy rechallenge, the panel agrees that 6 months is the minimum yet sufficient timeframe for rechallenge in case of disease progression after 2-year ICI treatment, although this is not possible in Italy to date. The Panel also agrees that in patients with PD-L1 ≥ 50% and progressing to immunotherapy, it would like to be able to continue ICI treatment beyond progression by adding platinum-based CT, as carried out in the Empower-lung 01 trial [85] and other ongoing trials, although this approach is not available in Italy. In the case of NSCLC oligoprogression after immunotherapy, combining local ablative therapies (namely radiotherapy, surgery, and radiofrequency ablation) with ICI continuation beyond progression appears to be safe and to provide promising long-term survival benefits. However, despite the encouraging data, most of these studies are small retrospective analyses, and larger prospective studies are needed [86].

Regarding immune-related toxicities, the recurrence rate of the same irAE associated with the discontinuation of ICI therapy after rechallenging with the same ICI is almost 30%, with colitis, hepatitis, and pneumonitis being the most common [87]. In this perspective, although a known risk of recurrence, the Panel agreed on resuming immunotherapy in case of severe endocrinological and cutaneous toxicity after resolution or improvement to grade (G) 1; it also agreed on not resuming ICI in case of severe pulmonary and muscular/cardiac toxicity, even in case of resolution. Of the several possible irAEs, myocarditis and pneumonitis are, in fact, those portending the poorest outcomes, with deaths reported in more than one-third of the cases [64]. Consensus on resuming ICI in case of resolution or amelioration of hepatic and gastroenteric toxicities was not met, possibly because of their higher risk of recurrence [87]. Finally, in patients who suspended immunotherapy due to irAEs, G1/2 toxicities, the time passed since toxicity resolution (even if >1 year), and the type of irAEs seem to not be determinant in choosing to resume immunotherapy, as the Panel could not reach consensus. Of note, during the second round of voting, consensus was almost reached on the fact that ICI could be resumed in most clinical scenarios except for cardiac, neurological, and hospitalization-requiring toxicities.

## 5. Conclusions

The Delphi survey conducted among Italian oncologists has elucidated key areas of both consensus and uncertainty regarding the use of immunotherapy in the treatment of NSCLC. The findings highlight the critical role of molecular characterization in guiding first-line treatment decisions, the potential benefits of chemoimmunotherapy in specific patient subgroups, and the possibility of immunotherapy rechallenge. Several patients’ features and molecular drivers seem to not single-handedly dictate a preference for a treatment regimen over another. Of note, clinicians have expressed their willingness to use treatment regimens not (yet) foreseen by the Italian regulatory standpoint. These insights contribute to the evolving landscape of NSCLC treatment and offer a valuable framework for clinical practice in Italy. They also suggest future clinical research to resolve the issues where consensus was not met.

The authors acknowledge the limitations linked to the use of a Delphi study in this study: potential bias in initial input, the subjectivity of responses, possible bias in the selection of panellists, and difficulty in reaching a consensus in areas with a lack of high-level evidence. They are aware that recommendations issued from such studies need further demonstrations in clinical studies.

## Figures and Tables

**Table 1:** Agreement/disagreement levels and consensus of the statements on NSCLC first-line therapeutic choice in the first and second rounds of the survey. High disease burden: sum of the longest diameter of target lesions ≥10 cm or a primary tumor with a diameter of ≥10 cm, LDH ≥ 2 ULN, presence of hepatic involvement, lymphangitis, pericardial effusion, brain involvement that cannot be addressed with local techniques, or an oncological emergency; low disease burden: absence of high burden; NGS: next-generation sequencing; “fit” patient: patients with PS ECOG 0-1 without significant comorbidities.

First Round				Second Round			
	Level of Disagreement	Level of Agreement	Consensus		Level of Disagreement	Level of Agreement	Consensus
In “fit” patients with PD-L1 < 50%, a combination of short-course chemotherapy (two cycles) and nivolumab + ipilimumab is preferred over chemotherapy + pembrolizumab in case of:				In “fit” patients with PD-L1 < 50%, high disease burden is a limitation for choosing a combination of short-course chemotherapy (two cycles) and nivolumab + ipilimumab.			
High disease burden	57.38%	6.56%	Not met		25.86%	58.62%	Not met
Low disease burden	8.20%	62.29%	Not met				
Regardless of the disease burden	32.79%	26.23%	Not met				
In “fit” patients with PD-L1 < 50%, a combination of short-course chemotherapy (two cycles) and nivolumab + ipilimumab is preferred over chemotherapy + pembrolizumab in the context of possibly receiving a platinum-based second line.				In “fit” patients with PD-L1 < 50%, a combination of short-course chemotherapy (two cycles) and nivolumab + ipilimumab is preferred over chemotherapy + pembrolizumab in the context of a platinum-based rechallenge in case of disease progression.			
	31.15%	44.26%	Not met		36.21%	32.76%	Not met
In patients with PD-L1 ≥ 50%, a high disease burden may prompt consideration, if possible, of chemoimmunotherapy combination treatment instead of anti-PD1/PD-L1 therapy as a single agent.				-	-	-	-
	4.92%	90.16%	Yes, consensus on agreement	-	-	-	-
Comprehensive molecular characterization (by NGS if available) of mutations should also be performed in all patients to optimize therapeutic choices regarding first-line chemotherapy and immunotherapy.				-	-	-	-
	0.00%	100.00%	Yes, consensus on agreement	-	-	-	-
In patients with PD-L1 ≥ 50%, first-line mono-immunotherapy treatment should be proposed in case of alterations in:				In patients with PD-L1 ≥ 50%, I would propose mono-immunotherapy versus platinum-based doublet chemotherapy in case of alterations in:			
*MET*	40.99%	47.54%	Not met	*MET*	44.83%	39.65%	Not met
*BRAF (V600)*	73.77%	14.76%	Not met	*BRAF* (V600)	31.03%	53.45%	Not met
*RET*	80.33%	13.12%	Yes, consensus on disagreement	-	-	-	-
*K-RAS* (G12C)	6.56%	83.61%	Yes, consensus on agreement	-	-	-	-
*HER2*	52.46%	22.95%	Not met	*HER2*	58.62%	27.59%	Not met
*NTRK*	78.69%	9.84%	Yes, consensus on disagreement	-	-	-	-
In patients with PD-L1 < 50%, first-line immunotherapy + chemotherapy treatment should be proposed in case of alterations in:				In patients with PD-L1 < 50%, I would propose immunotherapy + chemotherapy treatment over chemotherapy alone in case of alterations in:			
*MET*	27.87%	57.38%	Not met	*MET*	27.58%	58.62%	Not met
*BRAF* (V600)	65.57%	22.95%	Not met	*BRAF* (V600)	22.41%	67.24%	Not met
*RET*	62.30%	26.23%	Not met	*RET*	50.00%	43.10%	Not met
*K-RAS* (G12C)	4.92%	85.25%	Yes, consensus on agreement	-	-	-	-
*HER2*	40.99%	37.70%	Not met	*HER2*	34.48%	53.45%	Not met
*NTRK*	67.21%	19.68%	Not met	*NTRK*	48.28%	37.93%	Not met
In patients with PD-L1, <50% and candidates for first-line chemoimmunotherapy treatment, the presence of *BRAF* (V600) and/or *K-RAS* (G12C) mutations suggests treatment with:				In patients with PD-L1 < 50% and candidates for first-line chemoimmunotherapy treatment, the presence of *K-RAS* (G12C) mutations suggests a treatment with:			
Triplet (chemotherapy + pembrolizumab)	16.40%	49.18%	Not met	Triplet (chemotherapy + pembrolizumab)	3.44%	74.14%	Not met *
Quadruplet (chemotherapy + nivolumab + ipilimumab)	27.87%	27.87%	Not met	Quadruplet (chemotherapy + nivolumab + ipilimumab)	12.07%	63.79%	Not met
In patients with PD-L1 < 50% and candidates for first-line chemoimmunotherapy treatment, the presence of *MET*, *RET*, *HER2*, or *NTRK* mutations suggests treatment with:				In patients with PD-L1 < 50% and candidates for first-line chemoimmunotherapy treatment, the presence of *MET*, *RET*, *HER2*, or *NTRK* mutations suggests a treatment with:			
Triplet (chemotherapy + pembrolizumab)	22.95%	42.63%	Not met	Triplet (chemotherapy + pembrolizumab) for performing multiple cycles of chemotherapy	10.34%	72.41%	Not met
Quadruplet (chemotherapy + nivolumab + ipilimumab)	44.27%	22.95%	Not met	Quadruplet (chemotherapy + nivolumab + ipilimumab)	55.17%	18.96%	Not met
Q10. Co-mutation of *KRAS* with *STK11* +/- *KEAP1* is a criterion for preferring triplet over quadruplet.				Q8. In patients with PD-L1 < 50% and in the presence of *KRAS* co-mutation with *STK11 ± KEAP1*, first-line therapy choice depends on the efficacy data of anti-CTLA4.			
	22.95%	36.07%	Not met		15.52%	58.62%	Not met
In case of a “non-oncogene-addicted” tumor, PD-L1 expression ≥ 50%, and “never smoker” status, the first-line therapy choice is:				In case of a “non-oncogene-addicted” tumor, PD-L1 expression ≥ 50%, and “never smoker” status, the first-line therapy choice is:			
Immunotherapy	26.23%	63.94%	Not met	Immunotherapy	20.69%	74.14%	Not met *
Immunotherapy + chemotherapy (if possible, from a prescriptive standpoint)	9.84%	81.96%	Yes, consensus on agreement	-	-	-	-
Chemotherapy (without immunotherapy)	67.21%	18.03%	Not met	Chemotherapy (without immunotherapy)	62.07%	27.59%	Not met
Q12. In the case of a “non-oncogene-addicted” tumor, PD-L1 expression ≥ 90%, and “never smoker” status, the first-line therapy choice is:				Q10. In case of a “non-oncogene-addicted” tumor, PD-L1 expression ≥ 90%, and “never smoker” status, although the first-line therapy chosen is immunotherapy, I would consider immunotherapy + chemotherapy if it was possible from a prescriptive standpoint.			
Immunotherapy	9.84%	83.60%	Yes, consensus on agreement		10.35%	86.21%	Yes, consensus on agreement
Immunotherapy + chemotherapy (if possible, from a prescriptive standpoint)	16.40%	70.50%	Not met	-	-	-	-
Chemotherapy (without immunotherapy)	75.41%	14.75%	Yes, consensus on disagreement	-	-	-	-
In clinical practice, I use the cut-off of 10 mg/day prednisone (or equivalent dose) in therapeutic choice.				Beyond the regulatory aspect, exceeding the cut-off of 10 mg/day prednisone (or equivalent dose) in therapeutic choice is an absolute clinical contraindication to immunotherapy use.			
	24.59%	67.21%	Not met		82.76%	12.07%	Yes, consensus on disagreement
In patients with PD-L1 ≥ 50% and on steroid treatment with prednisone at >10 mg/day (or equivalent dose) for oncological needs (e.g., dyspnea, brain metastasis, other), the therapeutic choice is:				In patients with PD-L1 ≥ 50% and undergoing steroid treatment with prednisone at doses >10 mg/day (or equivalent dose) for oncological needs (e.g., dyspnea, brain metastasis, other), the treatment choice is:			
Immunotherapy	16.39%	75.40%	Yes, consensus on agreement	Immunotherapy	18.97%	72.42%	Not met *
Chemotherapy (without immunotherapy)	49.18%	32.79%	Not met	Chemotherapy (without immunotherapy)	43.11%	34.48%	Not met
-	-	-	-	Chemotherapy + immunotherapy, if possible, from a descriptive perspective	1.72%	94.83%	Yes, consensus on agreement

* Consensus almost met.

**Table 2:** Agreement/disagreement levels and consensus of the statements on NSCLC in special populations in the first and second rounds of the survey.

First Round				Second Round			
	Level of Disagreement	Level of Agreement	Consensus		Level of Disagreement	Level of Agreement	Consensus
In patients with PD-L1 < 50%, first-line treatment choice could be driven by the patient’s comorbidities (cardiac, renal, etc.) that influence their need for multiple cycles of chemotherapy, resulting in choosing the ipilimumab + nivolumab combination, which provides only two cycles of chemotherapy.				-	-	-	
	6.56%	88.53%	Yes, consensus on agreement	-	-	-	
Choosing a chemotherapy + immunotherapy combination depends on the presence of autoimmune diseases that, although controlled, would make the combination of two immunotherapies more hazardous and complex to manage than the anti-PD1 immunotherapy alone.				-			
	8.20%	85.25%	Yes, consensus on agreement	-	-	-	
In patients with PD-L1 < 50%, the choice of using triplet or quadruplet as first-line therapy is also affected by the presence of brain metastases.				In patients with PD-L1 < 50% and brain metastasis, the quadruplet would be preferred over the triplet.			
	16.40%	60.66%	Not met		10.34%	51.72%	Not met
In patients with PD-L1 < 50%, the choice of using triplet or quadruplet as first-line therapy is also affected by the presence of liver metastases.				In patients with PD-L1 < 50% and liver metastasis, the quadruplet would be preferred over the triplet.			
	31.15%	36.07%	Not met		27.58%	22.42%	Not met
In patients with PD-L1 < 50%, poor LIPI scores with high neutrophil/lymphocyte ratio and LDH value steer the therapeutic choice toward triplet versus quadruplet.				In patients with PD-L1 < 50%, the presence of poor LIPI score with high neutrophil/lymphocyte ratio and LDH value steer the therapeutic choice toward triplet versus quadruplet.			
	21.31%	40.98%	Not met		8.62%	51.73%	Not met
In clinical practice, the preference for the quadruplet over the triplet is based on the following factors:				In clinical practice, I prefer quadruplet over triplet in case of frail patients (e.g., advanced age (>75 years), performance status = 2, etc.).			
Advanced age (≥75 years)	34.43%	37.71%	Not met		44.83%	41.38%	Not met
Performance status = 2	40.99%	31.15%	Not met	-	-	-	
Non-adenocarcinoma histology	21.31%	54.10%	Not met	-	-	-	
PD-L1 negative (<1%)	16.40%	62.29%	Not met	-	-	-	
High disease burden	50.81%	18.04%	Not met	-	-	-	
Q21. In clinical practice, the preference for triplet over quadruplet is based on the following factors:				In clinical practice, I prefer quadruplet over triplet in case of non-adenocarcinoma histology and PD-L1 negative (<1%).			
Advanced age (≥75 years)	31.15%	31.15%	Not met		6.90%	81.04%	Yes, consensus on agreement
Performance status = 2	36.07%	32.79%	Not met	-	-	-	
Non-adenocarcinoma histology	50.82%	13.11%	Not met	-	-	-	
PD-L1 negative (<1%)	54.10%	18.03%	Not met	-	-	-	
High disease burden	11.48%	57.37%	Not met	-	-	-	

**Table 3:** Agreement/disagreement levels and consensus of the statements on NSCLC immunotherapy rechallenge in the first and second rounds of the survey.

First Round				Second Round			
	Level of Disagreement	Level of Agreement	Consensus		Level of Disagreement	Level of Agreement	Consensus
In clinical practice, after two years of treatment with first-line immunotherapy as a single agent, immunotherapy treatment should be discontinued in case of:				In clinical practice, after 2 years of first-line mono-immunotherapy treatment, discontinuation of immunotherapy treatment occurs in the case of:			
Complete response	8.20%	81.96%	Yes, consensus on agreement	-	-	-	-
Partial response	24.59%	57.37%	Not met	Partial response	20.69%	65.52%	Not met
Stable disease	39.35%	34.43%	Not met	Stable disease	43.10%	27.59%	Not met
Assuming it was possible, in case of progression after completion of 2 years with first-line immunotherapy, I would consider a rechallenge:				To perform a rechallenge with immunotherapy in case of disease progression after completion of 2 years of first-line immunotherapy treatment, the minimum time for disease control is:			
Yes, regardless of the time to progression	68.85%	29.51%	Not met	-	-	-	-
Yes, after 3 months	54.10%	24.59%	Not met	-	-	-	-
Yes, after 6 months	9.84%	78.69%	Yes, consensus on agreement	At least 6 months, if it was possible, from a descriptive perspective	10.34%	77.59%	Yes, consensus on agreement
Yes, after 1 year	0.00%	93.44%	Yes, consensus on agreement	At least 12 months, if it was possible, from a descriptive perspective	20.69%	60.34%	Not met
Yes, after a line of CT	22.96%	40.99%	Not met	-	-	-	-
In patients who discontinued the nivolumab + ipilimumab combination for G3 toxicity, continuing therapy with nivolumab alone can be considered.				In patients who discontinued the nivolumab + ipilimumab combination for G3 toxicity, continuing therapy with nivolumab alone can be considered if the toxicity resolves.			
	22.95%	68.85%	Not met		12.06%	84.49%	Yes, consensus on agreement
Assuming it was possible, in patients with PD-L1 ≥ 50% progressing to immunotherapy, I would like to continue immunotherapy beyond progression by adding platinum-based chemotherapy.				-	-	-	-
	6.56%	86.88%	Yes, consensus on agreement	-	-	-	-
Q26. In case of severe toxicity (≥G3) from immunotherapy with symptom resolution or improvement to G1, I would resume immunotherapy treatment in case of toxicity was:				Q21. In case of severe toxicity (≥G3) from immunotherapy with symptom resolution or improvement to G1, I would still not resume immunotherapy treatment in case the toxicity was:			
Pulmonary	75.40%	11.48%	Yes, consensus on disagreement	-	-	-	-
Gastroenteric	37.70%	36.07%	Not met	Gastroenteric	37.93%	50.00%	Not met
Endocrinological	3.28%	90.17%	Yes, consensus on agreement	-	-	-	-
Cutaneous	11.48%	81.97%	Yes, consensus on agreement	-	-	-	-
Hepatic	50.82%	40.99%	Not met	Hepatic	31.03%	58.62%	Not met
Cardiac/muscle	88.53%	0.00%	Yes, consensus on disagreement	-	-	-	-
In patients who have discontinued immunotherapy treatment due to toxicity, I would resume treatment at the time of disease progression, with close clinical monitoring:				In patients who have discontinued immunotherapy treatment for toxicity, I would resume treatment at the time of progression, with close clinical monitoring:			
Always	63.94%	21.31%	Not met	Always	48.28%	36.20%	Not met
Always, except in cases of cardiac or neurological toxicities or those that have required hospitalization	26.23%	70.49%	Not met	Always, except in cases of cardiac or neurological toxicities or those that have required hospitalization	18.97%	72.41%	Not met
At least 3 months after the resolution of toxicity	32.79%	27.87%	Not met	Only in the case of G1-2 toxicity	24.14%	63.79%	Not met
At least 6 months after the resolution of toxicity	29.51%	40.99%	Not met	-	-	-	-
At least 12 months after resolution of toxicity	29.51%	44.27%	Not met	-	-	-	-
In case of severe toxicity (≥G3) from immunotherapy, it is advisable to consult the organ specialist (if available)				-	-	-	-
	1.64%	96.72%	Yes, consensus on agreement	-	-	-	-

## Data Availability

The original contributions presented in the study are included in the article, further inquiries can be directed to the corresponding author.
